# Hyaluronic acid: emerging roles and biomaterial innovations in Alzheimer’s and Parkinson’s disease therapy

**DOI:** 10.3389/fphar.2026.1772172

**Published:** 2026-03-23

**Authors:** Yishu Wang, Jingwei Duan, Lixuan Zang, Ting Sun, Huimin Lv, Fei Liu, Xiaodong Ma

**Affiliations:** 1 Key Laboratory of Brain, Cognition and Education Sciences, Institute for Brain Research and Rehabilitation, Guangdong Key Laboratory of Mental Health and Cognitive Science, Ministry of Education, South China Normal University, Guangzhou, China; 2 Shandong Academy of Pharmaceutical Sciences, Engineering Research Center for Sugar and Sugar Complex, National-Local Joint Engineering Laboratory of Polysaccharide Drugs, Key Laboratory of Carbohydrate and Glycoconjugate Drugs, Jina, China; 3 National Glycoengineering Research Center, Shandong University, Qingdao, China; 4 School of Pharmaceutical Sciences, Cheeloo College of Medicine, Shandong University, Jinan, China

**Keywords:** Alzheimer’s disease, hyaluronic acid, hydrogels, nanoparticles, Parkinson’s disease

## Abstract

Hyaluronic acid (HA) is a key component of the extracellular matrix (ECM). Owing to its anti-inflammatory properties, biocompatibility and ability to contribute to ECM remodeling, HA is considered a promising therapeutic candidate for neurodegenerative diseases. This review summarizes the application of HA to treat Alzheimer’s disease (AD) and Parkinson’s disease (PD) and outlines the current understanding of the mechanism of action and strategies for HA-based biomaterial modification. For AD, HA is involved in several mechanisms including stabilizing the perineuronal net, reducing the toxic effects of Aβ and hyperphosphorylated tau, and modulating neuroinflammation through CD44/RHAMM signaling pathways. HA-based nanoparticles and hydrogels enhance drug delivery across the blood-brain barrier, facilitate Aβ clearance, and enable sustained, controlled release of therapeutic agents. In PD, HA regulates autophagic flux, inhibits α-synuclein propagation, and remodels the ECM to protect dopaminergic neurons. Modifications such as HA hydrogels with neurotrophic factors improve cell transplantation outcomes, while conjugates enhance mitochondrial targeting and dopamine delivery. While numerous preclinical studies have shown promise, significant challenges remain, including the high variability of HA formulations, limited blood-brain barrier penetration efficiency, and a paucity of well-designed clinical trials to validate preliminary findings. Future directions include standardizing laboratory protocols, developing hybrid systems integrating vascular endothelial growth factor and gene therapy, and adopting a patient-specific approach that leverages HA’s multi-targeted effects on the nervous system.

## 1 Introduction

**FIGURE 1:**
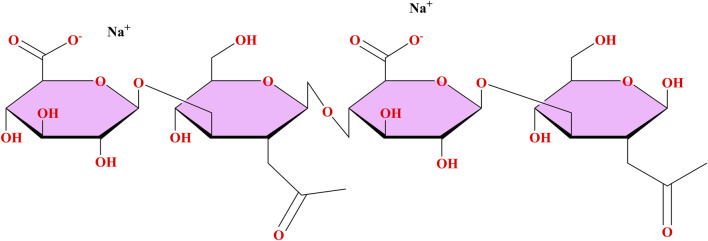
Chemical structure of HA.

Hyaluronic acid (HA) is a naturally occurring glycosaminoglycan present within the extracellular matrix (ECM) of multiple human tissue types, including the CNS (Jensen et al., 2020; Martin-Saldaña et al., 2025). The disaccharide unit of HA consists of D-glucuronic acid and N-acetyl-D-glucosamine (Salwowska et al., 2016). It is produced by three different hyaluronan synthases (either HAS1, HAS2 or HAS3) and subsequently degraded by various hyaluronidases (HYAL) (Palenčárová et al., 2025). This constant recycling maintains an optimal level of HA in the ECM and also exerts moderate control over tissue hydration, viscosity, and cell-cell communication (Vasvani et al., 2020). Within the brain, HA helps maintain a healthy balance of ECM materials, helps regulate the movement of neurons and promotes synaptic plasticity; additionally, HA plays an important role in the formation of perineuronal nets (PNN), which are vital for protecting neurons from injury and controlling the process of inflammation (Jia et al., 2022; Silva Garcia et al., 2019). Further, when HA is used as a drug delivery system or in regenerative therapy, the biocompatibility, biodegradability, and receptor binding capability of HA with CD44 and RHAMM, provides a unique platform for further research and clinical translation into the treatment of neurodegenerative disorders in which ECM dysregulation may be playing a significant role in exacerbating the underlying pathology (Zhang et al., 2021; Zhu et al., 2020). It is anticipated that future studies utilizing HA as a scaffold, either alone or with other materials, such as nanoparticles or hydrogels, will ultimately increase the ability to specifically target drug therapy and subsequently improve the effectiveness of drug therapy, thus providing greater patient benefits (Iaconisi et al., 2023) (Figure 1).

Alzheimer’s disease (AD) is the most common neurodegenerative disease, affecting over 50 million people around the world, and it is characterized by a progressive decline in cognitive function, memory loss and changes in behavior (Se Thoe et al., 2021; Soria Lopez et al., 2019). Pathophysiologically, AD is characterized by Aβ accumulation as well as aggregation of hyperphosphorylated tau into neurofibrillary tangles and chronic neuroinflammation, ultimately causing synaptic and neuronal dysfunction (Twarowski and Herbet, 2023; Wang and Colonna, 2019). In addition to synaptic dysfunction and neuronal degeneration, ECM dysregulation also acts on synapses by impairing the integrity of normal perineuronal nets and increasing the permeability of the blood-brain barrier (BBB), thereby contributing to oxidative stress and proteotoxic aggregates (Yang et al., 2024). Furthermore, vascular components associated with comorbid vascular dementia further exacerbate the neurodegenerative processes in AD, as evidenced by correlations between elevated HA levels in the cerebrospinal fluid (CSF) and BBB integrity markers (Takousis et al., 2019; Zetterberg, 2015). Current symptomatic AD treatments, e.g., cholinesterase inhibitors, do not provide a therapeutic strategy to halt disease progression and highlight the importance of developing novel therapeutic strategies based on ECM remodeling and neuroprotection (Lista et al., 2024; Wang et al., 2025).

Parkinson’s disease (PD) is the second most common neurodegenerative disorder. Approximately 10 million people worldwide are affected by PD, which primarily causes motor symptoms (bradykinesia, tremor, and rigidity), but it also includes non-motor symptoms (constipation and cutaneous manifestations) (Sveinbjornsdottir, 2016). At a cellular level, PD is characterized by a selective loss of dopaminergic neurons from the substantia nigra, aggregation of α-synuclein into Lewy bodies and mitochondrial dysfunction, which, in turn, lead to impaired autophagy, oxidative stress and neuroinflammation (Lotankar et al., 2017; Raza et al., 2019). HA-mediated ECM reorganization in PD alters the diffusion of substances through the extracellular space and subsequently influences the response of glial cells, potentiating α-synuclein propagation (Soria et al., 2020). Currently, levodopa is the treatment of choice for managing the symptoms of PD, but because levodopa works for only a limited time, patients taking levodopa often experience ‘motor fluctuations’ and many side effects (Tambasco et al., 2018). This highlights the urgent need to develop new disease-modifying therapies that target the underlying biological processes associated with PD.

The intersection of HA with AD and PD pathologies lies in its multifaceted roles in ECM stabilization, anti-inflammatory signaling, and neuroprotection, thereby offering novel therapeutic avenues (Damodarasamy et al., 2020; Diaz-Lasprilla et al., 2024). In AD, HA inhibits Aβ-induced neurotoxicity and tau pathology by serving as a scaffold to maintain PNN structural integrity; reduces neuroinflammation by preserving PNN structural integrity via preserving high-molecular-weight HA (HMW-HA) within the ECM (Dong et al., 2023; Reed et al., 2019). Similarly, in PD, HA modulates a variety of cellular processes that affect autophagy regulation; reduces oxidative stress *via* protection from neuroinflammatory cytokines; and remodels the ECM to enhance clearance of toxic agents in genetically-modified and symptomatically-treated non-human animal models (Rahman et al., 2020). In addition, HA-based formulations have been developed, such as HA nanoparticles and HA hydrogels, which facilitate enhanced delivery of drugs to the CNS as compared to conventional methods due to using the aforementioned properties of HA to specifically target the location of drug action and have sustained release properties (Chen et al., 2023; Zhang et al., 2022).

This review comprehensively synthesizes research progress on hyaluronic acid (HA) for the management of Alzheimer’s disease (AD) and Parkinson’s disease (PD), linking mechanistic insights to the application of advanced biomaterials. As a narrative review, it is based on a targeted search of relevant literature in databases such as PubMed and Google Scholar, focusing on key terms including “hyaluronic acid”, “Alzheimer’s disease”, “Parkinson’s disease”, “nanoparticles”, and “hydrogels”, without adopting a formal systematic review protocol. The review points out not only the role HA can play in both disorders but also its significance as a reference point for researchers, clinicians, and pharmacologists who seek to develop integrated therapies. Subsequent sections of this review discuss the specific roles and modifications of HA in AD and PD, culminating in a discussion of conclusions and future perspectives aimed at supporting translational research.

## 2 HA in AD

### 2.1 The role of HA in AD

The potential targeting of HA for treatment of AD is based on its role in stabilizing the ECM, thus contributing to neurodegeneration. It has been shown in scientific literature that HA contributes to the regulation of Aβ by regulating Aβ toxicity, tau pathology, and endolysosomal function. Studies have demonstrated that patients with vascular dementia, have increased levels of HA present in their cerebrospinal fluid (CSF) (Nägga et al., 2014). It is believed that this represents a compensatory mechanism for the vascular injury/damage sustained by the individual. The increased level of HA is also associated with a greater degree of increase in BBB permeability, as demonstrated by the greater CSF/serum albumin ratio. Therefore, HA may also serve as a potential biomarker for defining the contribution of vascular injury to cognitive impairment.

To clarify the age-related trajectory of HA in relation to AD: HA abundance follows a dynamic pattern across the lifespan. At birth and during early development, the brain contains high levels of HMW-HA, which supports neural plasticity, cell migration, and ECM organization (Bignami et al., 1993). As individuals age from adolescence through adulthood, HA levels progressively decline under physiological conditions, reaching a steady-state equilibrium in healthy adults (Reed et al., 2019). This age-related decrease in HA is particularly pronounced in elderly populations and correlates with diminished ECM integrity, increased astrocyte reactivity, and heightened susceptibility to neurodegeneration (Fowke et al., 2017). Importantly, low HA abundance during normal aging serves as a trigger for astrogliosis; in aged rodents and humans, declining HA concentrations are associated with increased expression of astrocytic markers (GFAP, S100β) and pro-inflammatory gliosis, creating a permissive environment for neurodegenerative processes (Cargill et al., 2012). However, in AD, a paradoxical secondary rise in total HA levels occurs. This elevation reflects a pathological compensatory response rather than restoration of healthy ECM homeostasis. Critically, the increased HA in AD is predominantly composed of LMW-HA fragments generated through aberrant ECM remodeling and hyaluronidase activity. These LMW-HA fragments, unlike their HMW counterparts, actively promote neuroinflammation by activating TLR4 pathways in microglia and astrocytes, stimulating the release of pro-inflammatory cytokines such as TNF-α and IL-1β, and exacerbating Aβ aggregation and tau pathology (Taylor et al., 2007). Thus, while low HA in aging predisposes to glial activation and neuronal vulnerability, the high abundance of pro-inflammatory LMW-HA in AD perpetuates chronic inflammation and accelerates disease progression (Reed et al., 2019). Building on this, evidence shows that the new molecular characteristics of HA promote their effects biologically in relation to AD. HMW-HA has some anti-inflammatory effect on neurons while low molecular weight (LMW)-HA may promote neuroinflammation. In AD mouse models and human patients, inflammation is mainly associated with LMW-HA. LMW-HA fragments during extracellular matrix (ECM) remodeling and promotes the production of pro-inflammatory cytokines such as tumor necrosis factor-α (TNF-α). In contrast, HMW-HA exerts protective anti-inflammatory effects, though the exact chain length threshold linked to inflammatory responses remains unclear in both rodents and humans (Reed et al., 2019; Dong et al., 2023). Neuroinflammatory processes in the brain regions affected by AD indicate elevated levels of HA accumulation correlate with other neuropathological changes such as, amyloid plaques and tau tangles as seen in post-mortem tissue studies showing high levels of HA in severely affected AD individuals compared to non-affected individuals (Reed et al., 2019). The increase in expression of HA Synthase 2 (HAS2) and Tumor Necrosis Factor Stimulated Gene 6 (TSG6) seen in late-stage AD increase stabilization of HA and could therefore be involved in providing a degree of protection for neurons from oxidative stress-induced cell death in the advanced stages of AD. TSG6 plays a critical role in stabilizing endogenous HA in the brain by catalyzing the formation of covalent HA-heavy chain complexes, which enhances HA’s structural integrity and anti-inflammatory properties. While not strictly essential for the integration of HA nanoparticles or hydrogels, TSG6 may facilitate better ECM incorporation in therapeutic designs by promoting cross-linking (Reed et al., 2019). Therapies targeting the enhancement of higher molecular weight HA could potentially provide protection against ECM reorganization that increases the presence of TAU pathology. ECM disorganization induced by tau pathology disrupts PNNs, resulting in disrupted synaptic plasticity. An example of the implications of such a finding is that when TAU undergoes hyperphosphorylation this results in displacement of axonal-localized HAS-1, thereby resulting in synthesis of shorter chain HA via increased expression of HAS3 inhibitory to the formation of PNNs, thus resulting in a greater vulnerability of neurons (Li et al., 2017). Therefore, from a therapeutic perspective restoring the function of HAS1 may preserve the structural integrity of PNNs and offer a means to enhance the resilience of neurons.

Differences between men and women further complicate HA’s function, but they also offer the possibility for more individualized approaches to therapy based on a person’s sex. Women with AD have lower levels of HA in their CSF than men, which seems to correlate with differences in their respective inflammatory markers, perhaps helping to explain the higher AD prevalence in women when compared to men (Nielsen et al., 2011). It has been suggested that this lower level of HA may be due to the impact of estrogens on HA metabolism and that these lower levels of HA may contribute to the increased risk of developing both vascular and inflammatory pathologies among women. Interestingly, HA appears to protect neurons from Aβ1-42 neurotoxicity in some cell culture systems via interactions with the PNNs and via glycosaminoglycans of the chondroitin sulfate family (Miyata et al., 2007). Furthermore, it has also been shown that removing these chains with chondroitinase ABC will result in no protection being afforded by the HA, thus providing support to the idea that HA has a structural role in providing ECM-mediated protection. However, chondroitinase ABC (ChABC) also degrades HA at physiological brain pH, and injections into the brain often lead to HA breakdown, potentially minimizing the observed ‘protective’ effects of HA post-ChABC treatment; moreover, PNNs require HA as a backbone for assembly, and without HA, complete PNN formation is unlikely, leading to impaired neuronal protection (Miyata et al., 2007; Dong et al., 2023). These findings support the idea that interventions using HA, such as exogenously providing HA, should be developed to support the PNNs and reduce Aβ aggregation, especially when considering gender stratification in the design of clinical trials.

In recent years, new knowledge has emerged showing how HA is related to treatment of AD. The mechanism for the connection between HA and AD has been studied recently, especially with regards to the hyaluronidase (HYAL)-CD44 axis. In AD animal models, Aβ and hyperphosphorylated tau proteins bind to the subunits of the vacuolar ATPase resulting in defective acidification of the endolysosomal compartment which promotes neurodegeneration (Kim et al., 2023). Conversely, HYAL overexpression through either enzyme activity or CD44 signaling downregulates the degradation of HA thereby allowing restoration of lysosomal function and clearance of proteotoxic aggregates. Treatment with HYAL improves recall memory deficits in 3xTg-AD mice suggesting that manipulating the turnover of HA provides a means to rescue neuronal proteostasis. An additional discovery indicates that certain non-ubiquitinated HAS1 mutant proteins can translocate to the nuclear speckle domain. This suggests possible regulatory pathways for both gene transcription and ECM remodeling during the progression of AD (Zhang et al., 2024). Disruption of HA synthesis through the AβPP-tau-HAS1 pathway suggests that targeting either the ubiquitination of HAS1 or improving the stability of HAS1 may potentially be a means to address both neurodegenerative changes in AD through promoting healthy levels of HA.

In astrocyte models, HA degradation influences reactivity, with reduced HA content in multi-interpenetrating polymer networks enhancing reactive phenotypes like GFAP and S100β expression (Munoz-Pinto et al., 2019). This relationship between HA degradation and astrocyte reactivity mirrors the age-related decline in HA observed *in vivo*. Specifically, in aged rodents (>18 months) and elderly humans (>65 years), brain HA concentrations decrease by approximately 30%–50% compared to young adult levels, particularly in hippocampal and cortical regions vulnerable to AD pathology (Cargill et al., 2012). This age-dependent HA depletion correlates strongly with increased expression of astrocytic activation markers (GFAP, S100β, vimentin) and pro-inflammatory mediators, establishing low HA as a trigger for age-related astrogliosis (Munoz-Pinto et al., 2019). Notably, in AD mouse models (APP/PS1, 3xTg-AD) and postmortem human AD tissue, regions with minimal residual HA exhibit the most pronounced astrogliosis and microglial activation, whereas areas retaining higher HMW-HA show relatively preserved neuronal integrity (Cargill et al., 2012; Reed et al., 2019). Thus, the age-related decline in HA creates a pro-inflammatory ECM environment that predisposes aged brains to exacerbated neurodegeneration when AD pathology emerges.

Microglia-HA interactions further modulate the inflammatory landscape in aged and AD-affected brains. Activated microglia express multiple hyaluronidases (HYAL1, HYAL2, HYAL3) that enzymatically cleave HMW-HA into LMW-HA fragments (<200 kDa) (Stern et al., 2006). These LMW-HA fragments function as damage-associated molecular patterns (DAMPs), binding to pattern recognition receptors—primarily TLR2, TLR4, and CD44—on microglial and astrocytic surfaces (Taylor et al., 2007). TLR4 engagement by LMW-HA activates NF-κB and MAPK signaling cascades, driving transcription of pro-inflammatory cytokines (TNF-α, IL-6, IL-1β), chemokines (CCL2, CXCL10), and reactive oxygen species (ROS) (McKee et al., 2019). This creates a feed-forward inflammatory loop: microglial hyaluronidase activity generates more LMW-HA, which in turn amplifies microglial activation and HA degradation (Garantziotis and Savani, 2019). Although the precise stoichiometry and kinetics of this process remain incompletely defined—particularly the relative contributions of individual HYAL isoforms and the threshold fragment sizes for maximal TLR activation—the overall mechanism is well-established (Stern, 2004). Notably, in AD, this LMW-HA-driven inflammation coexists with elevated total HA levels, creating a paradoxical state where high total HA masks a shift toward pro-inflammatory LMW species (Reed et al., 2019). Thus, microglia do not merely passively respond to HA; they actively remodel the HA landscape, converting anti-inflammatory HMW-HA into pro-inflammatory LMW-HA, thereby perpetuating the chronic neuroinflammation characteristic of AD.

These findings indicate that HA scaffolds could be strategically engineered to regulate glial responses in AD by restoring HMW-HA content while limiting LMW-HA accumulation, thereby reducing chronic inflammation and supporting neuronal survival. Importantly, the increased total HA levels observed in AD represent a double-edged phenomenon: the initial elevation (primarily driven by HAS2/TSG6 upregulation) may transiently buffer against vascular injury and provide neuroprotection in early disease stages. However, as AD progresses, dysregulated ECM remodeling shifts the HA pool toward pro-inflammatory LMW fragments. This long-term accumulation of LMW-HA, rather than HMW-HA, contributes to a pathological profibrotic ECM environment, impairs synaptic plasticity, accelerates Aβ plaque formation, and sustains microglial/astrocytic activation (Fowke et al., 2017). Thus, therapeutic strategies must distinguish between promoting HMW-HA synthesis/stabilization and inadvertently increasing LMW-HA burden. Overall, HA’s therapeutic value must be considered in conjunction with an optimal therapeutic approach to maintain optimal levels of HA, potentially by using HAS inhibitors and/or enhancing HYAL activity. The findings suggest that HA has potential therapeutic applications and warrants additional preclinical testing and development of optimal delivery techniques, including nanoparticle encapsulation of HA for access through the BBB to provide targeted neuroprotection.

### 2.2 HA-based modifications for AD treatment

#### 2.2.1 HA-based nanoparticles

Because of its biocompatibility, biodegradability, and ability to target the overexpressed CD44 receptors found on inflamed tissues, including the brain during neurodegenerative disease processes, HA has attracted substantial interest from the nanomedicine community (Grieco et al., 2022; Janzen et al., 2022). In the specific context of AD, nanoparticles made from HA represent an exciting opportunity as a drug delivery vehicle because HA’s hydrophilicity enhances nanoparticle stability and facilitate their passage through the BBB, as well as reduce neuroinflammation (Lei et al., 2021; Tracy et al., 2023; Wu et al., 2022). Furthermore, these HA-based nanoparticles can encapsulate therapeutic agents, inhibit the aggregation of Aβ, scavenge ROS, and protect against neuronal injury, thus providing solutions to several of the major pathological hallmarks associated with AD.

A significant advancement in the area of AD research involves the creation of magnetic mesoporous silica nanoparticles that are called HA-MMSN-1F12. HA-MMSN-1F12 nanoparticles are capable of inducing depolymerization of insoluble Aβ plaques into soluble Aβ oligomers and nanoparticles. In addition, upon intravenous administration, HA-MMSN-1F12 nanoparticles cross the blood-brain barrier, do not undergo significant hepatic uptake, and facilitate the elimination of captured A-beta directly through intestinal metabolism, therefore reducing Aβ burden in the brain, neuroinflammation, and cognitive deficits without causing any hepatic or renal toxicity (Liu et al., 2022). Notably, the use of co-assembled chitosan-hyaluronic acid nanoparticles cross-linked with glutaraldehyde (CHG NPs) as a theragnostic agent to both detect and inhibit the Aβ protein fibrillation has emerged. These biomass-derived nanoparticles have unique cluster-triggered emission properties, which allow them to be used for fluorescence-based probing with high sensitivity of Aβ oligomers and fibrils down to 0.1 nM detection limits. *In vitro* studies have demonstrated that the CHG NPs selectively bind with negatively charged Aβ aggregates and inhibit fibril formation at concentrations ≥360 μg/mL. *In vivo* validation in *C. elegans* AD models has shown that they can be used to image Aβ plaques and prevent deposition of the protein, and have demonstrated HA as a key contributor to electrostatic attraction and inhibition of aggregation (Wang et al., 2021). Therefore, CHG NPs demonstrate dual function, both in being a diagnostic tool for imaging, and as a therapeutic tool that inhibits the formation of Aβ fibrils, presenting a unique set of capabilities. However, scaling of production and ensuring prolonged bio-distribution are some issues that have yet to be resolved.

When comparing the age-related differences between the presence of AD and the use of HA-conjugated nanoparticles, the following studies were conducted using poly (lactic-co-glycolic acid)-block-HA nanoparticles that bind CD44 on activated astrocytes and microglia. In aged APP/PS1 mice, the 200 nm HA-conjugated nanoparticles accumulate in the hippocampus after an intravenous injection and transport of the nanoparticles occurs through an increase in BBB permeability due to aging and AD pathology (Tracy et al., 2023). Most HA nanoparticle studies use LMW-HA for easier nanoformulation and improved penetration, though some incorporate HMW-HA for enhanced stability. Nanoparticle accumulation occurs primarily in the extracellular space rather than within neurons, driven by factors such as age-related decreased clearance and increased BBB leakiness, which promote retention in inflamed regions (Tracy et al., 2023). In contrast, young controls show no significant accumulation of the nanoparticles in their brains, indicating that increased vascular permeability due to the aging process is an important mechanism for transporting the nanoparticles to the brain. This study suggests that HA’s targeting specificity for neuroinflammatory cells may enhance therapeutic retention in AD patients, but additional pharmacokinetic studies are needed to assess potential toxicity in non-AD elderly patients. Importantly, improved central nervous system exposure in diseased models may reflect BBB disruption rather than the intrinsic transport capacity of the delivery platform. Thus, interpretations of such studies must cautiously distinguish between disease-induced changes in BBB permeability and active nanoparticle-mediated transcytosis, to avoid potentially overestimating nanoparticle efficacy in scenarios with an intact BBB. Ulteriorly, the use of microfluidic fabrication techniques in conjunction with 3D printing has provided new means of precisely engineering nanoparticle-based platforms for targeted drug delivery. For example, with microfluidics, investigators have been able to create nanoparticles made of chitosan/hyaluronic acid nanoparticles loaded with rivastigmine. In these studies, the investigators developed processes that influenced how uniformly rivastigmine is distributed throughout the nanoparticle and were able to produce nanoparticles with sizes ranging from 309.9 to 476.36 nm and tailored sustained-release profiles. In some cases, the initial release of rivastigmine was observed to occur very quickly within ∼15 min after injection of the nanoparticles, followed by sustained release over 24 h, and the extended release of rivastigmine was attributed to electrostatic forces between chitosan/hyaluronic acid and the rivastigmine molecule (Atashbasteh et al., 2025). This type of fabrication technique provides research opportunities for using staggered herringbone micro-mixers in 3D-printed microfluidic devices to ensure reproducible and scalable production of nanoparticles and overcome poor uniformity associated with traditional fabrication methods. This research highlights the potential for using HA to encapsulate hydrophilic drugs used to treat symptoms of AD and suggests that there may also be potential to customize dosing in the clinical setting.

**FIGURE 2:**
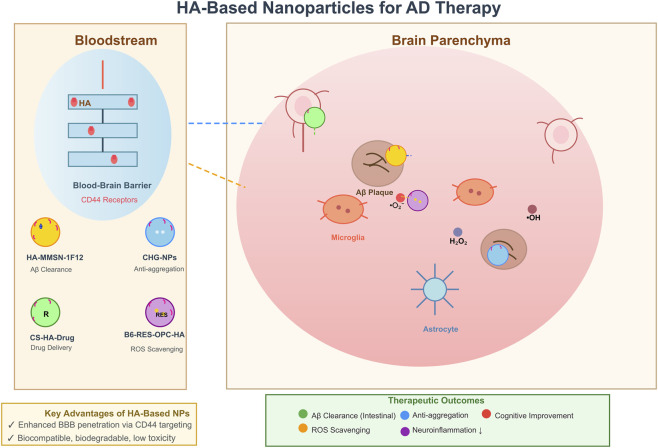
Schematic of HA-based nanoparticles for AD therapy: BBB transcytosis and intracerebral therapeutic actions. Note: In the bloodstream, HA-based nanoparticles cross the BBB via CD44 receptor-mediated transcytosis, with tailored functionalities (Aβ clearance, anti-aggregation, drug delivery, ROS scavenging). Within the brain parenchyma, these nanoparticles target AD-associated components to exert therapeutic effects—including Aβ clearance, anti-aggregation, cognitive improvement, ROS scavenging, and reduced neuroinflammation. Microglia are depicted interacting with nanoparticles, potentially digesting HA fragments to modulate inflammation, though this process amplifies LMW-HA release and pro-inflammatory signaling.

Collectively, these investigations underscore the versatility of HA-based nanoparticles in AD therapy, from Aβ disaggregation and ROS neutralization to diagnostic imaging and controlled drug release. The primary benefits of HA nanoparticle use are through increasing bioavailability, decreasing toxicity, and targeting delivery, which may cause an evolution in the way AD is treated through prevention. Despite these advantages, there are limitations, including potential immunogenicity, variability in BBB penetration among AD patients, and the need for long-term efficacy trials, as well as continued evaluation of these HA nanoparticle products. Negative or neutral results also merit attention—for example, variable enhanced permeability and retention (EPR) effects across studies, which can lead to inconsistent biodistribution, and batch-to-batch variability in nanoparticle size and drug loading that may compromise reproducibility. Additionally, further studies or clinical trials of HA hybrid products that combine HA and gene therapy/neuroimaging agents will provide for the continued success of HA nanoparticles as a way to fight against the multiple pathologies of AD (Figure 2).

#### 2.2.2 HA-based hydrogels

HA is proving to be a highly adaptable biomaterial for the fabrication of hydrogel-based systems used to treat AD (Simpson et al., 2020). HA’s unique characteristics, including its biocompatibility, biodegradability, and ability to form stable hydrogels, make it an excellent candidate for the delivery and release of therapeutic drug molecules, and promoting successful interactions with neuronal tissues (Schuurmans et al., 2021). As a multifactorial disease, AD’s progression is driven by a variety of factors such as the aggregation of Aβ peptides, neuroinflammation, and cholinergic deficits (Chen et al., 2024). HA-based hydrogels offer a multifaceted platform to combat many of the underlying causes of neurodegeneration associated with AD and therefore may inhibit the formation of Aβ fibrils, enable sustained therapeutic drug delivery to the brain, and facilitate improvement through imaging for theranostic approaches. Recent studies have successfully utilized HA’s hydrophilic properties and binding affinity for the CD44 receptor that is present at higher levels in inflamed areas of the brain to develop new hydrogel systems, yielding promising *in vitro* and *in vivo* results.

A key function of the system is to combine HA with natural agents that prevent Aβ aggregation, thus creating HA-based nanogels that can self-assemble. An example of this is how curcumin, a polyphenolic compound that has been shown to possess anti-amyloid effects but which has low solubility in water, was covalently linked to HA (Kabir et al., 2023; Maiti et al., 2018). The resulting nanogel outperformed free curcumin as it was able to provide enhanced inhibition of Aβ aggregation and self-assemble into hydrogels that were tunable from 100 to 300 nm in diameter based on substitution efficiency. These formulations provided an isolating effect through preventing Aβ monomer-monomer interactions. The hydrophobic binding and electrostatic repulsion mechanisms also lead to changes in Aβ conformation. *In vitro* studies demonstrated that when curcumin is substituted at 5%–10%, fibrillation of Aβ40 and Aβ42 was reduced by 50%–60% using thioflavin T fluorescence and decreased cytotoxicity against SH-SY5Y neuroblastoma cells from 60% viability to over 80% viability (Jiang et al., 2016). The multifunctional properties of HA highlight the importance of HA as both a carrier and active modifier of Aβ; its negative charges repel Aβ peptides that carry similar charges, thus directing the formation of off-pathway aggregates that are less toxic to neurons. Building on this, dual-modification strategies have amplified HA hydrogel efficacy by incorporating synergistic inhibitors. The efficacy of HA hydrogel has been enhanced through the use of epigallocatechin-3-gallate (EGCG) and curcumin to form “Nano-Gels” with distinctly different “Hydrophobicity” values (Jiang et al., 2018). This allows for even finer tuning of the Nanostructure. The resulting Nano-Gels have been shown to have stable size (150–200 nm) and zeta potential (−30 mV) due to having 4%–5% substitution of EGCG and 2%–3% substitution of curcumin. The increase in stability to bind Aβ has been confirmed through thioflavin T assays that demonstrated 60%–70% reduction in the aggregation of Aβ42, relative to that obtained from single-inhibitor systems. The synergy of EGCG and curcumin occurs because EGCG preferentially binds to unfolded Aβ Monomers, while curcumin binds preferentially to Fibril Forms of Aβ. The HA Hydrogel creates a network of hydrogels that isolates Aβ and stretches Aβ in opposite directions through hydrophobic and electrostatic forces. As a result, the Co-Action of the inhibitors is hypothesized to block the formation of Fibrils and/or restructure current fibrils, thereby potentially reversing early-stage AD pathology (Jiang et al., 2018). However, this optimal ratio of the EGCG and curcumin inhibitors suggests the need for a delicate balance between the two. As the level of substitution increased, the nanostructure became compacted and the accessibility and efficacy decreased.

Theranostic applications further expand HA hydrogels’ utility by integrating diagnostic elements. The use of the iron oxide nanoparticle incorporated into the HA nanogel through *in situ* thiolation, created nanoparticles that are sized between 120 and 150 nm with superparamagnetic characteristics (Chen X. et al., 2022). The incorporation of iron oxide nanoparticle within the hydrogel HA nanoparticles resulted in the inhibition of Aβ aggregation by approximately 40%–45% and also promote the disaggregation of fibrils by 10%–15% at 10 mM concentrations. Through the use of MRI studies *in vitro*, the T2/T2 contrast was shown to be enhanced significantly, which would provide for non-invasive monitoring of AD. The Cytotoxicity testing performed on Astrocytes showed a high level of biocompatibility (>95% at 100 μg/mL) and the level of plasma protein adsorption was less than due to the negative surface charge. These two functions will assist in addressing two of the main challenges in diagnosing Aβ in relation to dementia by providing a means of detecting Aβ early in the course of Alzheimer’s disease while also targeting the underlying cause of the disease. As such, there needs to be *in vivo* validation of the ability of the system across the BBB and the long-term risks of accumulation of Iron in the body (Chen X. et al., 2022).

Another form of innovative therapeutic delivery of HA-modified hydrogels for brain-targeted AD therapy is through intranasal application. Citicoline sodium (CIT), which is a choline precursor that acts as a choline donor and supports the creation of acetylcholine and the repair of cellular membranes, was encapsulated within HA-modified nano-transbilosomes (AbouElhassan et al., 2022; Guan et al., 2025). CIT-loaded nano-transbilosomes are produced from an optimized formulation with an average size of 178 nm and an average entrapment efficiency of 75%. When subjected to *ex vivo* testing, the thermogel system allows for sustained nasal delivery of CIT at a concentration of 500 μg/cm^2^. Research using rats with AD causes using aluminum chloride has shown that this system reduces expression levels of hippocampal Aβ1-42 by 50%–60% compared to controls and decreases levels of malondialdehyde and NF-κB, while also increasing expression levels of miR-137. In the intranasal delivery of these HA-modified nanoparticles, nanoparticle distribution throughout the brain (e.g., via olfactory pathways to the hippocampus) correlates with reduced neuropathology. This suggests a primarily direct effect, where diffused nanoparticles locally reduce Aβ and inflammation; however, indirect effects may also occur. Signaling in nanoparticle-rich regions could induce anti-inflammatory changes in adjacent, nanoparticle-sparse areas (AbouElhassan et al., 2022). Using HA as a delivery vehicle increases the targeting of the blood-brain barrier through CD44/RHAMM receptors and increases the amount of CIT that is taken to the brain. This form of administration also avoids the effects of metabolism by the liver; however, in addition to the issues pertaining to the mucociliary clearance mechanism and efficiency, there remain many opportunities for additional process improvements.

Overall, these findings indicate that HA hydrogels may transform our ability to manage AD by not only inhibiting the progression of the disease but also allowing for targeted delivery and imaging. The mechanism of isolation and conformational modulation will result in improved results relative to free drugs, which may provide a means to help delay the progression of AD.

**FIGURE 3:**
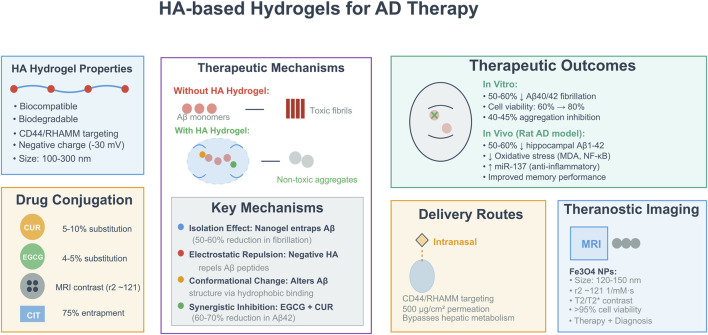
Schematic of HA-based hydrogels for AD therapy: properties, mechanisms, and therapeutic outcomes. Note: HA hydrogel converts toxic Aβ aggregates to non-toxic ones via four key actions (isolation effect, electrostatic regulation, conformational change, synergistic inhibition of EGCG + CUR). *In vitro* (Aβ40/42 fibrillation inhibition, cell viability) and *in vivo* (AD rat model: Aβ aggregation inhibition, hippocampal Aβ1-42 reduction, oxidative stress attenuation, improved memory) effects.

However, these studies also highlight ongoing limitations, including variability in the *in vivo* degradation rates of HA hydrogels, variable EPR effects that may inconsistently influence hydrogel retention across models, and inconsistent intranasal delivery outcomes in some studies due to factors such as mucociliary clearance. Negative findings, such as neutral effects on Aβ aggregation in high-substitution variants or batch-to-batch variability in gel stiffness, underscore the need for standardization. There is also a need for future studies to examine both the safety and efficacy of HA hydrogels during clinical testing in human subject populations. Subsequently, additional studies using hybrid systems, such as combining gene therapy with inhibitor therapies, are needed to fully utilize HA’s unique capabilities and to move toward the development of personalized treatments for AD (Figure 3).

#### 2.2.3 Other HA modifications

There have been advances in the characterization of HA modifications on different nanoscale systems that have shown promise to help alleviate the classical hallmarks of AD, including the aggregation of Aβ, hyperphosphorylation of tau, neuroinflammation, and cognitive deficits.

An example of this is in the use of HA-doped nanotransfersomes as a method of intranasal drug delivery for an AD medication known as donepezil hydrochloride (DPZ), which inhibits the action of cholinesterases, enzymes that break down acetylcholine, and is frequently used to alleviate symptoms of AD (Zhang and Gordon, 2018). The use of an HA-based formulation provides improved stability and mucoadhesion properties of the transport system, allowing the transport system to avoid gastrointestinal side effects of oral DPZ administration. An optimized DPZ-HA-nanotransfersome formulation had a vesicle size of 227.5 nm, entrapment efficiencies of 75.83%, and sustained release of 37.94% over 8 h, as well as significant permeation of 547.49 mg/cm^2^ through the nasal mucosa within 24 h (Salem et al., 2024). In addition, DPZ biodistribution studies in a rat model indicated that the transport system had a 5.08 drug targeting index, an efficiency of 508.25%, and 80.32% of the drug was delivered directly from the nose to the brain via the olfactory pathways, which confirms that HA increases drug absorption via the olfactory pathway (Salem et al., 2024). For intranasal delivery, nanoparticle distribution (e.g., to the cortex and hippocampus) directly reduces local pathology such as Aβ plaques, but indirect effects are also apparent. Nanoparticle-induced signaling (e.g., reduced NF-κB in treated regions) spreads to adjacent regions, thereby enhancing overall cognitive function (Salem et al., 2024). Safety evaluations resulting from histological studies and cognitive assessments indicate that this route of delivery may allow for prolonged non-invasive use of DPZ, thereby minimizing peripheral side effects and allowing for extended periods without re-dosing; these findings will need to be further evaluated before use in human clinical applications for the intranasal treatment of AD.

In another study, metal-organic frameworks (MOFs) were coupled with HA, and hafnium-based MOFs (Hf-MOFs) were used to entrap rhynchophylline that has both anti- Aβ and anti-tau features (Lin et al., 2025). HA@Rhy@Hf-MOF nanoparticles are consistently sphere-shaped with a negative surface charge for stability, sustained release of rhynchophylline, and improved rhynchophylline solubility and penetrability into the brain when compared to the traditional formulation of rhynchophylline. This is attributed to the fact that both the Hf-MOF’s and HA’s properties reduced the low solubility of rhynchophylline and allowed for better penetration into the brain through the BBB. When tested in Aβ-induced AD mouse models, HA@Rhy@Hf-MOF treatments improved Morris Water Maze performance, novel object recognition, and elevated plus maze tests while also decreasing hippocampal Aβ plaque formation and tau hyperphosphorylation through immunofluorescence and Western blot analysis (Lin et al., 2025). These results suggest that HA enhances the ability of the Hf-MOF system to cross the BBB more effectively and also targets inflammation by interacting with CD44 receptors to increase uptake via endocytosis. This modification of Hf-MOF exploits its ability to generate singlet oxygen and thus to modulate oxidative stress and has a synergistic effect with HA in assessing and treating multifactorial AD pathology; however, the possibility of metal ion accumulation should be monitored with the prolonged use of Hf-based compounds.

HA-modified carbon quantum dots (CQDs) containing verapamil (VRH), an anti-inflammatory/calcium channel blocker, have been evaluated in in vitro models of amyloidosis-induced AD (Mosalam et al., 2024). HA-CQD/VRH had an 81.2% association rate, and resulted in superior neuroprotection in SH-SY5Y/Neuro-2a cells compared with unformulated VRH pretreatment. The use of HA-CQDs to load VRH prior to treating cells significantly decreased Aβ-induced generation of reactive oxygen species, inflammation, and reduced mitochondrial dysfunction and enhanced cytochrome P450 enzymes, cytochrome c oxidase, CREB-regulated transcriptional coactivator 3, and proliferative indices. By modifying the surfaces with HA to facilitate stable transport in serum to nervous tissue and offer target specificity, the incorporation of HA allows for increased modulation by HA-CQD/VRH on calcium homeostasis and CREB activation (Mosalam et al., 2024). The combination of CQD’s optical characteristics with the targeting properties of HA holds promise for expanding imaging capabilities and represents a step towards theranostic development; however, further studies must validate the effectiveness of HA-CQD/VRH through BBB penetration and investigate the long-term compatibility of HA-CQD/VRH with biological systems due to potential risks of aggregation of CQDs.

In summary, the modifications made to HA resulted in new structures with improved delivery to the brain by increasing the amount of drug delivered over a longer period of time. This makes HA a key material for AD nanotherapeutics. Among various methods of non-invasively delivering drugs into the body, both the use of intranasal routes or conjugate forms of drugs are appealing, while combining drugs into either MOF or CQD increase the number of different uses for HA. In addition, ongoing challenges include scaling production, obtaining regulatory approval, and conducting clinical trials to demonstrate the safety and efficacy of new composite compounds in humans. Negative or neutral results include variable EPR effects in MOF-based systems, inconsistent intranasal delivery outcomes due to patient-specific nasal anatomy, and batch-to-batch variability in CQD association rates that may hinder scalability. Future studies should investigate the potential of utilizing combination therapies with multiple agents in combining the best features of HA to address the myriad of impacts exhibited from AD, including developing new possibilities to innovate in the area of pharmacology.

## 3 HA in PD

### 3.1 The role of HA in PD

PD is a neurodegenerative disease characterized by the progressive loss of dopaminergic neurons in the substantia nigra, resulting in both motor impairments and non-motor symptoms (Kempster and Ma, 2022; Otero-Losada et al., 2022). HA has several advantageous properties that make it an excellent option for use in drug delivery systems, cell transplantations, and as modulators of neuroinflammation. Many studies have shown that HA can relieve some of the symptoms associated with the disease and also modulate the underlying pathologies (Havránková et al., 2025; Vacca et al., 2022), suggesting that it has the potential to affect the progression of the disease through its interactions with cellular pathways and the local extracellular environment. To date, most of these applications are still in their infancy.

At the mechanistic level, research on the genetics of Parkinson’s disease has revealed that HA induces the occurrence of abnormal activation of intracellular degradation via the autophagy pathway in cells associated with the pathology of photoreceptor degeneration (Dong et al., 2023; Laffleur et al., 2015). Mutations in the VPS35 genes D620N mutation and other familial PD genes, related to the HMW-HA pathway, cause excessive levels of HMW-HA to increase the activation of HA and therefore decrease the rate of autophagy in a study using cell lines and animal models created through transgenic lines (Rahman et al., 2020). There were also several significant upregulation pathways including the ECM-receptor, and the PI3K-AKT pathways, as found through RNA sequencing. These findings suggest that HMW-HA creates a barrier to the autophagic pathway and thus inhibits autophagy, and HMMR knockdown of HMW-HA receptor rescues autophagic inhibition, showing that the inhibition of autophagic flux by HMW-HA and targeting of the HMW-HA pathway may restore cellular clearance mechanisms that are impaired in Parkinson’s disease. The excessive accumulation of HMW-HA may also promote the aggregation of proteins, such as the accumulation of alpha-synuclein, by inhibiting lysosomal degradation of cellular debris due to the excessive levels of HMW-HA (Rahman et al., 2020). Modulation of HA levels may be a potential novel strategy for enhancing autophagic flux and slowing disease progression, however, data from human clinical studies are currently not available to confirm this. The current work also suggests that the previously accepted concepts of the beneficial role of HA in health are limited. HMW-HA appears to exacerbate the pathology of mutants, which indicates that HA has different effects depending on the cellular context in which it is present.

Further supporting HA’s neuroprotective potential, a recent study conducted on a mouse model of α-synuclein neurodegeneration using electron microscopy and single nanotube tracking, the depletion of HA caused changes in the organization and diffusion of brain ECS (Soria et al., 2020). Following the inoculation with α-synuclein, enlarged ECS dimensions and increased nanoscale diffusion were observed in the substantia nigra, which correlated to the loss of HA matrix surrounding reactive microglia (Soria et al., 2020; Wu et al., 2024). The use of hyaluronidase to deplete HA acutely or chronically resulted in reduced dopaminergic cell death and α-synuclein load, as well as an increase in microgliosis and ECS diffusivity (Soria et al., 2020). Therefore, it appears that HA serves as a structural organization and barrier in the ECS and has a role in the propagation of α-synuclein. The depletion of HA remodeling of ECM after HA depletion may aid in clearing out toxic aggregates, thus providing a means of modifying the illness. However, HA depletion is associated with an inflammatory response, raising concerns about the long-term safety of this approach (Altman et al., 2019). Thus, careful modulation is required to avoid excessive glial activation.

Overall, the current evidence strongly supports HA as a multifaceted therapeutic agent for PD. Although pilot studies and mechanistic studies have shown that HA could potentially be used to treat PD, the current studies have been limited by small sample sizes and the focus on animal models, necessitating further clinical trial validation to confirm the efficacy of HA as a treatment for PD. There is a need for more research on HA preparation for CNS delivery, either through intranasal routes or via nanoparticle systems, in order to use HA to its full potential in preventing the progression of PD.

### 3.2 HA-based modifications for PD treatment

#### 3.2.1 HA-based hydrogels

HA hydrogels are made from a naturally occurring glycosaminoglycan that has a high concentration in the extracellular matrix of neural tissues (Burdick and Prestwich, 2011; Pérez et al., 2021). As a result, HA hydrogels can deliver cells and/or growth factors and/or therapies to help overcome some of the difficulties faced in PD therapy including low cell survival following implantation, limited neuronal differentiation and continued inflammation (Grieco et al., 2022; Ouyang et al., 2023). The initial studies showed that HA systems increased the viability of neural progenitors and allowed for the incorporation of neurotrophic factors that could be used to promote dopaminergic neuron regeneration.

One study provides foundations for a novel HA hydrogel which could be used to carry genetically engineered BDNF (brain-derived neurotrophic factor) to neural cells (Nakaji-Hirabayashi et al., 2009). The HA chains were formed by crosslinking the chains to histidine-tag polypeptides using Zn^2+^-chelation technology, enabling the stable incorporation of BDNF into the hydrogel. *In vitro* experiments demonstrated that when neural cells are seeded into this BDNF-hydrogel, they exhibit significantly increased viability compared to controls that do not contain BDNF at 3 days post-seeding and remain bound for at least 12 days. The use of HA hydrogels appears to offer a way to enhance cell survival rates in PD animal models through sustained delivery of neurotrophic support. This may ultimately translate into improved results for therapeutic approaches that involve cell transplants to replace the lost dopaminergic neurons. However, it remains to be determined whether similar positive outcomes will be observed using BDNF-infused HA hydrogels over longer periods of time and whether these BDNF-infused hydrogels stimulate normal integration and functional recovery when implanted in the brain.

Building on this, research on tunable mechanical properties of HA hydrogels has demonstrated how they can influence the fate of neural progenitor cells (NPCs), and are therefore important in applications for PD. Different degrees of methacrylate modification were used to achieve hydrogels with compressive moduli that match those of the neonatal brain and adult spinal cord, respectively (Seidlits et al., 2010). Soft hydrogels, like the stiffness of neonatal brains, primarily supported the differentiation of NPCs encapsulated in those hydrogels into neurons that were β-III tubulin-positive and had elongated processes, suggesting further maturation of those neurons. Conversely, hydrogels with increased stiffness promoted differentiation of NPCs towards astrocytes, evidenced by the expression of glial fibrillary acidic protein. Primary astrocytes only adopted a spread morphology in the stiffest variants of the hydrogels. Overall, these results support that HA hydrogels provide mechanical cues that guide NPC lineage commitment, with softer matrices more favorable for neuronal outcomes that mimic early development environments and increase the yield of dopaminergic neurons within PD grafts. Therefore, the tunability of HA hydrogels offers a means of optimizing hydrogel design to specific stages of PD; however, effective clinical translation will require both understanding the mechanics and the variability of human tissue and the immune response associated with that tissue.

Additional developments have incorporated mesenchymal stem cells (MSCs) with the utility of the MSCs’ secretome within HA hydrogels. The MSC, derived from adipose tissue, was embedded within a hydrogel that was crosslinked with polyethylene glycol and demonstrated the ability to release factors that protect dopaminergic cells from the toxicity caused by 6-hydroxydopamine (6-OHDA), which is commonly used as an animal model for PD. Additionally, the 3D hydrogel environment significantly improved the viability and paracrine activity of the MSCs when compared to a 2D culture format. Therefore, based on the results of these studies, the authors propose that the spatial confinement of the MSCs increases their ability to secrete paracrine factors that may play a role in alleviating the symptoms associated with PD by reducing oxidative stress and inflammation rather than through direct replacement therapy (Chierchia et al., 2017). However, further studies regarding the *in vivo* effectiveness and the identification of key mediators within the secretome are warranted for targeted therapies development.

Several recent studies have been investigating the combination of HA hydrogels and glial cell line-derived neurotrophic factor with the use of human embryonic stem cell derived midbrain dopaminergic neurons to increase long-term post-transplant survival. Additionally, HA hydrogels were engineered to allow for optimal controlled release and dispersion of cells during their implantation into the rodent models of PD. Engineered HA hydrogels significantly increased the viability of midbrain dopaminergic neurons that were encapsulated within the gel and transplanted into rodent models of PD (Adil et al., 2017). Furthermore, cell-instructive HA biomaterials co-delivering mDA neurons with survival and dispersion factors ameliorated Parkinsonism in rodent animal models and allowed for improved dispersion, integration and dopamine release from graft sites. Behavioral improvements in this case were observed in amphetamine-induced rotation tests and linked with increased neuronal survival, highlighting the potential importance of hydrogel-mediated cues to overcome barriers to transplantation (Adil et al., 2018). These results suggest that multifunctional HA platforms have the potential to increase the effectiveness of cell replacement therapy for PD, but remaining issues such as immunogenicity and long-term factor release profiles must first be resolved prior to commencement of clinical studies in humans.

**FIGURE 4:**
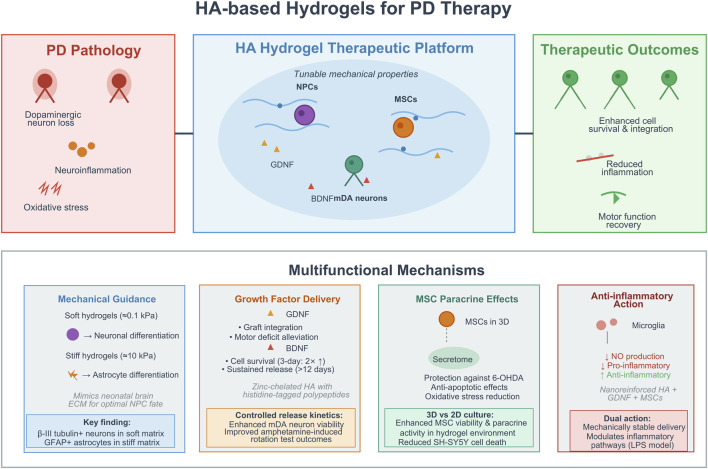
Schematic of HA-based hydrogels for PD therapy: platform, mechanisms, and therapeutic outcomes. Note: key components: PD pathology (dopaminergic neuron loss, neuroinflammation, oxidative stress); the HA hydrogel therapeutic platform (tunable mechanical properties, loaded with MSCs, GDNF, and iDOPA neurons); therapeutic outcomes (enhanced MSC survival/integration, reduced inflammation, motor function recovery); and multifunctional mechanisms (mechanical guidance for neuron differentiation via soft/stiff hydrogels; controlled growth factor (GDNF/BDNF) delivery to improve cell viability; MSC paracrine effects via secretome-mediated neuroprotection; anti-inflammatory action by regulating microglia and inflammatory pathways).

In summary, HA hydrogels provide a multifaceted platform that serves as a scaffold for PD treatment by delivering cells, maintaining growth factors, and providing mechanical support to promote neuronal repair. However, variability in degradation rates and the need for personalized mechanical properties represent opportunities for improvement. Negative findings include inconsistent enhanced permeability and retention (EPR) effects in cell-laden hydrogels, neutral outcomes in stiff variants that favor astrocyte over neuronal differentiation, and batch-to-batch variability in crosslinking that may compromise mechanical consistency. Future research should focus on developing long-term, scalable *in vivo* applications of HA hydrogels, in addition to combining HA hydrogels with next-generation technologies such as gene editing, to achieve the best therapeutic potential (Figure 4).

#### 3.2.2 Other HA-Based modifications

Recent studies have explored HA conjugates for buccal administration, nanoparticle-based mitochondrial targeting, and dopamine-mimicking applications, revealing key insights into their potential for PD management.

Notably, thiolation of HA to form hyaluronic acid-cysteine ethyl ester (HAC)—via amide bond formation between HA and L-cysteine ethyl ester using coupling agents (e.g., EDAC and NHS)—has yielded a mucoadhesive carrier for rotigotine, a dopamine agonist used in PD treatment (Laffleur et al., 2015). The results of several studies show that HAC has many advantageous characteristics compared to HA such as being approximately 1.49 times more stable, 3.47 times more swelling capacity, and approximately 12.16 times more mucosal adhesion to porcine mucosa. In addition, HAC has been shown to increase the permeability of rotigotine by 1.18 times across mucosal barriers resulting in a sustained release profile while reducing gastrointestinal degradation of rotigotine. Thus, HAC addresses the issues faced by many PD patients, namely problems related to fluctuating drug levels and dysphagia by providing non-invasive buccal delivery that avoids the first-pass effect and enhances patient compliance. The enhanced mucosal adhesion observed with HAC is attributed to the formation of disulfide bonds between the thiol groups present on HAC and the cysteine-rich mucus components highlighting thiolation as a potential method for optimizing polymer-based drug delivery systems.

There is also another means to use HA in repairing mitochondria, and that is the concept of using HA-containing nanoparticles (HA-NPs) having various molecular weights as a means of targeting mitochondrial injury because mitochondrial injury is a characteristic of the pathological process associated with PD. HA-NPs having a molecular weight of 2,190 kDa were shown to protect the mitochondria of SH-SY5Y neuroblastoma cells and PINK1 knockout MEFs from injury by improving mitochondrial function (i.e., increasing production of ATP, decreasing levels of reactive oxygen species). After the restoration of mtDNA integrity and an increase in cellular viability due to mild reversible injuries, HA-NPs exhibit the ability to function as an antioxidant agent (Chen Y. et al., 2022). In cases of irreversible mitochondrial injury, HA-NPs are modified with USP30 siRNA and PINK1 antibodies to selectively enhance mitophagy. PINK1 antibodies allow selective targeting of damaged mitochondria by promoting the accumulation of PINK1 on the outer membrane of the damaged mitochondria under certain cellular stresses, allowing recruitment of Parkin through the knockdown of USP30 (Chen Y. et al., 2022). The combined use of both of these strategies has shown to significantly improve symptoms of mitochondrial dysfunction in animal models of PD, as demonstrated by a reduction in apomorphine-induced rotational behaviors in mice that had undergone 6-OHDA lesions. Overall, this project provides a better understanding of the potential use of HA as a nanocarrier for gene therapy in neurodegenerative diseases.

One notable modification involves the utilization of HA as a ligand for dopamine has yielded a new class of substances in which dopamine is covalently conjugated to HA to produce essentially HA modified with dopamine (DA-HA). These compounds have properties similar to DA, but because they do not degrade or oxidize spontaneously, they also have reduced toxicity. DA-HA was produced using EDC/NHS chemistry and has been shown to bind to and elicit the same responses as DA in astrocytes expressing GRABDA2m and also in *ex vivo* calcium imaging. However, while DA and other synthetic dopaminergic drugs such as L-DOPA or 6-OHDA can undergo autoxidation leading to the generation of highly ROS that can be deleterious to neurons, DA-HA does not show evidence of auto-oxidation for 48 h post preparation (Kim et al., 2024). In addition, in animal studies, DA-HA demonstrated less dopaminergic neuronal loss than 6-OHDA following injection into the striatum. In models of PD induced by 6-OHDA, supplementation with DA-HA was associated with improved motor function as the rotation asymmetry produced by the 6-OHDA was abolished. Importantly, the reduced autoxidative activity of DA-HA may be due to the stabilizing influence of the HA polymer backbone that would slow the rate of degradation of DA-HA and thus minimize the potential formation of free radicals and oxidative stress, which has been a limiting factor for the use of dopaminergic substances for the treatment of PD.

Overall, these changes demonstrate how HA can be used to treat PD, including ways to improve drug penetration and adhesion and how to deliver dopamine directly into mitochondria. Still, several challenges remain: determining the optimal molecular weight for maximum brain penetration, demonstrating long-term biocompatibility in human clinical trials, and developing efficient large-scale HA synthesis methods to yield a low-cost product. Additional negative results include variable EPR effects in mitochondrial-targeted NPs, inconsistent buccal delivery outcomes due to mucosal variability, and batch-to-batch differences in thiolation efficiency that can affect adhesion consistency. It will be beneficial for future research to combine all of these strategies together to create hybrid multifunctional HA products comprising mucoadhesive, neuroprotective, and dopaminergic properties; this would allow for further advancements in providing individualized interventions for treating PD.

## 4 Conclusion

The potential of HA as a versatile agent in the treatment of AD and PD is demonstrated in this review of the latest research. Both AD and PD share several key pathological features, such as disrupted ECM homeostasis, inflammation of the nervous system, and the death of neurons. The combination of HA’s ability to regulate neuroinflammatory processes, modify amyloid-beta toxicity, and remodulate the ECM of the brain, as well as the ability of advanced materials such as hydrogels, nanoparticles, and conjugates to improve drug delivery and support neuroprotection, allows for HA to provide therapeutic benefits for a range of symptoms associated with cognitive and motor dysfunction. In addition to the benefits of HA itself, HA-modified therapies have also been shown to overcome several obstacles associated with traditional pharmaceutical therapies, such as crossing the BBB and providing sustained release of HA. However, to balance these promising findings, it is crucial to acknowledge key limitations: variable EPR effects across different delivery platforms, inconsistent outcomes in intranasal and buccal delivery due to interindividual biological variability, and batch-to-batch inconsistencies that could impede clinical translation. As such, this review offers new insights into HA’s ability to aid in the development of new paradigms of disease-modifying treatments, and the need for integrated strategies to exploit these properties of HA to promote neuronal repair and lessen the negative impacts of pathological processes, leading to more effective and targeted treatments for patients with AD and PD.

## 5 Future perspectives

While there have been some advances in researching HA’s role in AD and PD, there exist many limitations preventing full possible translation of the research. The existing evidence has primarily come from *in vitro* studies and animal trials with very few if any robust evidence from human clinical trials to establish the true efficacy and safety of HA on diverse populations with various comorbidities such as vascular dementia and those patients who are in advanced stages of PD. Another limiting factor in the research is the variability of HA formulations (i.e. molecular weight, degradation rates, conjugation methods), thus limiting reproducibility. Theoretical benefits of HA such as anti-inflammatory effects may vary based on differences in formulation (i.e. higher molecular weight HA may provide anti-inflammatory benefits; whereas lower molecular weight HA may potentially increase the inflammatory response). Due to the lack of clinical trials, the potential for long-term biocompatibility of HA also remains poorly defined and of particular concern. Such concerns include the potential for creating an immune response, or developing an unintended accumulation over time in tissue that are not considered to be targeted for treatment, or create off-target effects (i.e. fibrotic responses) in the delicate microenvironment of the brain.

To address these limitations, including the variable EPR effects, inconsistent intranasal/buccal delivery outcomes, and batch-to-batch variability noted in preclinical studies, collaborative efforts are needed to conduct comprehensive clinical validation. This should start with Phase 1 and Phase II clinical studies focused on the safety and pharmacokinetic characteristics of drugs in humans before conducting more advanced clinical studies. Utilizing biomarkers (for example, levels of hyaluronic acid in cerebrospinal fluid) will allow investigators to better track the effectiveness of treatments, thus bridging the gap between preclinical studies and clinical application of treatments in humans. Utilization of advanced delivery methods for hyaluronic acid will improve the delivery of these therapies to the brain. Technologies such as nano-engineered delivery systems will further optimize delivery of these drugs through the blood-brain barrier without sacrificing drug stability. Thus, we will improve the efficiency of targeting and delivering treatments to the human brain.

Of note, combining HA with emerging modalities can also help deal with potential mechanical deficiencies. Combining HA with gene therapy and/or CRISPR-based gene editing will enable precise regulation of HA synthesis and degradation by targeting hyaluronan synthases and hyaluronidases. Furthermore, combining HA with gene therapy/CRISPR-based strategies will enable you to address the pathological profile of an individual based upon things such as gender or mutated genes. Finally, using a patient-specific approach based upon a multi-omics approach, you can move beyond using HA on a standalone basis and create synergistic treatment regimens that include HA based scaffolding, which can enhance the uptake of Neurotrophic Factors and Anti-Inflammatory Agents. Therefore, it creates a more holistic approach to treating the complexities associated with the issues of neurodegeneration associated with AD and PD. Ultimately, this type of collaboration among pharmacologists, neuroscientists and bioengineers to standardize protocols and accelerate these innovations will allow HA to transition from a supportive biomaterial to one of the primary methods of addressing neurodegenerative diseases.
